# Data-Driven Identification of Biomarkers for In Situ Monitoring of Drug Treatment in Bladder Cancer Organoids

**DOI:** 10.3390/ijms23136956

**Published:** 2022-06-23

**Authors:** Lucas Becker, Felix Fischer, Julia L. Fleck, Niklas Harland, Alois Herkommer, Arnulf Stenzl, Wilhelm K. Aicher, Katja Schenke-Layland, Julia Marzi

**Affiliations:** 1 Department for Medical Technologies and Regenerative Medicine, Institute of Biomedical Engineering, University of Tuebingen, 72076 Tuebingen, Germany; lucas.becker@uni-tuebingen.de (L.B.); katja.schenke-layland@uni-tuebingen.de (K.S.-L.); 2 Cluster of Excellence iFIT (EXC 2180) “Image-Guided and Functionally Instructed Tumor Therapies”, University of Tuebingen, 72076 Tuebingen, Germany; 3 Institute of Applied Optics (ITO), University of Stuttgart, 70569 Stuttgart, Germany; felix.fischer@ito.uni-stuttgart.de (F.F.); alois.herkommer@ito.uni-stuttgart.de (A.H.); 4 Mines Saint-Etienne, CNRS, UMR 6158 LIMOS, Centre CIS, Université Clermont Auvergne, 42270 Saint Jarez-en-Priest, France; julia.fleck@emse.fr; 5 Department of Urology, University of Tuebingen Hospital, 72076 Tuebingen, Germany; niklas.harland@med.uni-tuebingen.de (N.H.); arnulf.stenzl@med.uni-tuebingen.de (A.S.); 6 Center of Medical Research, Department of Urology at UKT, University of Tuebingen, 72076 Tuebingen, Germany; aicher@uni-tuebingen.de; 7 NMI Natural and Medical Sciences Institute at the University of Tueingen, 72770 Reutlingen, Germany

**Keywords:** patient-derived tumor models, personalized medicine, non-invasive molecular imaging, machine learning, drug efficacy testing

## Abstract

Three-dimensional (3D) organoid culture recapitulating patient-specific histopathological and molecular diversity offers great promise for precision medicine in cancer. In this study, we established label-free imaging procedures, including Raman microspectroscopy (RMS) and fluorescence lifetime imaging microscopy (FLIM), for in situ cellular analysis and metabolic monitoring of drug treatment efficacy. Primary tumor and urine specimens were utilized to generate bladder cancer organoids, which were further treated with various concentrations of pharmaceutical agents relevant for the treatment of bladder cancer (i.e., cisplatin, venetoclax). Direct cellular response upon drug treatment was monitored by RMS. Raman spectra of treated and untreated bladder cancer organoids were compared using multivariate data analysis to monitor the impact of drugs on subcellular structures such as nuclei and mitochondria based on shifts and intensity changes of specific molecular vibrations. The effects of different drugs on cell metabolism were assessed by the local autofluorophore environment of NADH and FAD, determined by multiexponential fitting of lifetime decays. Data-driven neural network and data validation analyses (k-means clustering) were performed to retrieve additional and non-biased biomarkers for the classification of drug-specific responsiveness. Together, FLIM and RMS allowed for non-invasive and molecular-sensitive monitoring of tumor-drug interactions, providing the potential to determine and optimize patient-specific treatment efficacy.

## 1. Introduction

Patient-derived tumor organoids have emerged as useful in vitro models for the high-throughput screening of drugs for individualized cancer treatment [1,2,3]. In vitro three-dimensional (3D) organoids, producible in a short time from small amounts of tissue, enable drug testing and the identification of potential diagnostic biomarkers. Such 3D organoids can be generated not only from surgical specimens, but also from endoscopic fine-needle aspirates, biopsy samples or even from patients’ body fluids [4,5], allowing for the recapitulation of a wide range of disease stages and clinical conditions. To date, the majority of preclinical bladder cancer research has been conducted with bladder cancer cell lines or mouse models [6,7], poorly representing the features of human bladder tumors. Recently, the generation of mouse and human bladder cancer organoids was reported [8,9], recapitulating the broad histopathological and molecular spectrum of human bladder cancer and suitable for analyses of patient-individualized drug responses. One of the main obstacles in generating organoids is obtaining the patient’s tissue, which is typically received from a biopsy. A novel, simplified and non-invasive method is to collect the urine of a patient, eliminating the need for surgery under anesthesia [4,10]. 

Drug metabolism and uptake, dose-dependent efficacy and the development of drug resistance are key determinants in the treatment of cancer patients. Within the last decade, it has been well recognized that these parameters vary greatly from patient to patient, necessitating personalized options for treatment [11,12,13]. Pretreatment screening of the dose-dependent potency of chemotherapeutics increases success rates and precludes drug overuse, thereby preventing side effects and additional patient suffering. Moreover, such patient-specific information could make therapies more cost-effective. 

Conventional methods to monitor drug-induced effects in cell cultures typically include either cell viability assays or gene and protein expression assays, which allow detailed analysis of total protein or DNA composition [14,15]. Overall, these methods assess the pooled response of a batch of organoids and ignore the cellular heterogeneity that drives resistance to tumor treatment while being time-consuming and requiring lysis of 3D organoids to extract biomolecules [16]. Spatially resolved techniques such as immunofluorescence imaging inherit other limitations such as sample fixation and the incorporation of a fluorophore that could interfere with native intracellular biochemistry. Moreover, each fluorophore probes only one specific biomolecule, limiting the amount of obtainable biochemical information. 

Advances in non-destructive imaging techniques have evolved over the past decade, providing time-resolved insight into cellular metabolism and molecular composition. Here, label-free fluorescence lifetime imaging microscopy (FLIM) utilizing the endogenous fluorescence of nicotinamide adenine dinucleotide (NADH) and flavin adenine dinucleotide (FAD) provides new possibilities for gathering information about metabolic pathways. Both coenzymes are involved primarily in the mitochondrial tricarboxylic acid cycle and electron transfer chain, where they are engaged in the formation of adenosine triphosphate and reactive oxygen species (ROS) as part of the energy metabolism and apoptosis pathway [17]. One hallmark of cancer cells is the reprogramed metabolism in their energy production. According to the “Warburg effect”, cancer cells are characterized by a dominating aerobic glycolysis metabolism compared to the normally favored oxidative phosphorylation [18]. FLIM possesses the fidelity to identify minute changes in the distribution of bound and free NADH and FAD as a direct measure of the metabolic state [19]. Hence, FLIM is a promising tool for monitoring the direct drug response of chemotherapeutics in organoid models [20].

Complementary to FLIM imaging, non-invasive Raman microspectroscopy (RMS) enables the marker-independent and molecular-sensitive identification and localization of subcellular structures. Molecular fingerprints of Raman active biomolecules such as proteins, lipids, or nucleic acids can reflect a specific tissue state or cellular phenotype [21,22,23,24].

These methods yield a large amount of data not describable by conventional univariate models. Therefore, data-driven machine learning tools such as principal component analysis (PCA), k-means clustering, or neural networks have been introduced for the analysis of RMS data to identify the main features of cellular changes after drug treatment in different and complementary approaches [25,26]. Whereas PCA and k-means methods are based solely on linear transformations, neural networks make use of non-linear activation functions and are able to find rather unintuitive and ulterior correlations in the data, which ultimately yields better clustering [27]. Another research-oriented goal is to utilize these machine learning tools to find new, hitherto unknown biomarkers with completely unbiased approaches.

In this study, 3D organoids of the bladder cancer cell line RT112, bladder cancer organoids (BCO) derived from primary tumor biopsy tissue, and urine-derived organoids (UCO) from bladder cancer patients were treated with cisplatin (*cis*) or venetoclax (*vtx*) and evaluated with FLIM and RMS. Herein, we aim to address the molecular and metabolic response of different organoid entities and to develop a multiparametric data-based model that can predict patient-specific treatment efficacies.

## 2. Results

We characterized three different organoid models derived from a bladder cancer cell line RT112, patient-derived primary bladder cancer tissue (BCO) as well as patient urine (UCO) and compared their direct reaction to anticancer drugs *cis* and *vtx*. RMS and FLIM were implemented for spatially and time-resolved measurements of cellular drug responses.

### 2.1. Raman Microspectroscopy Allows Label-Free Imaging of Bladder Cancer Organoids

**Figure 1:**
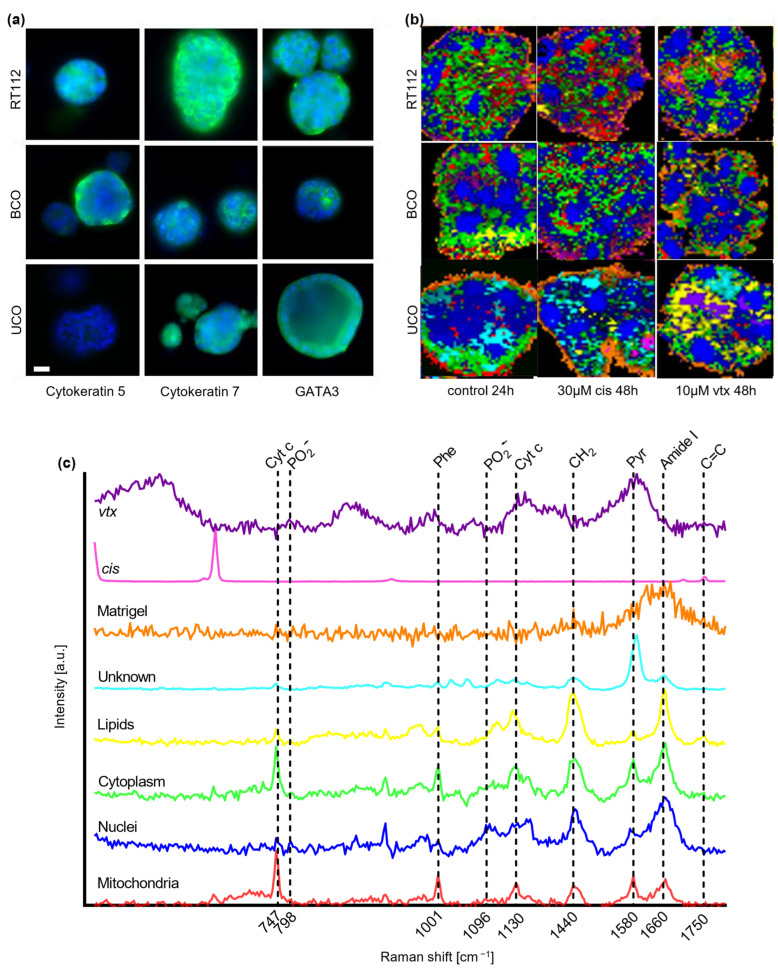
**Immunofluorescence (IF) and Raman microspectroscopy (RMS) of different bladder cancer organoid models.** (**a**) IF images of untreated RT112, BCO and UCO organoids stained for cytokeratin 5, cytokeratin 7 or GATA3 (green) and DAPI (blue). Scale bar: 25 µm. (**b**) True component analysis (TCA) images of RT112, BCO and UCO organoids. Displayed is one representative scan of untreated and treated organoids (30 µM cisplatin (*cis*) or 10 µM venetoclax (*vtx*)) for each organoid model. Scale bar: 20 µm. (**c**) Relevant TCA spectra for the identified cellular components.

Organoids were derived from a bladder cancer cell line, primary bladder cancer tissue and urine. Immunofluorescence (IF) staining for the epithelial cell markers cytokeratin 5 and cytokeratin 7 and the transcription factor GATA3 was performed to characterize the three cancer organoid models (Figure 1a). IF images served for validation of the cancerogenic phenotype of the cultured urothelial organoids and confirmed successful cultivation. All organoids expressed cytokeratin 7 and GATA3, whereas cytokeratin 5 was not expressed in UCO. 

Organoids were treated with different concentrations of *cis* or *vtx*. RMS was performed after 24 and 48 h, respectively. Utilizing true component analysis (TCA), six major cellular components were identified that could be attributed to mitochondria (red), nucleic acids (blue), cytoplasm (green), lipids (yellow), an unknown component (turquoise) and Matrigel (orange) based on their location within the organoids (Figure 1b) and their Raman fingerprint spectra (Figure 1c). To obtain information about the distribution of both drugs in the organoids, acquired reference spectra of solid drugs were included in the TCA calculation. In Raman images, *cis* is represented in pink, while *vtx* is presented in purple.

The assignment of the TCA components to their biological origins is based on the evaluation of individual peaks of the fingerprint spectra. Mitochondria were identified by their pronounced peaks at 747 and 1130 cm^−1^ reported for cytochrome c and b [28,29] and in-house measurement of a reference spectrum. Nuclei are assigned to the peak pair at 798 and 1096 cm^−1^, indicators for PO_2_ occurring in DNA [30], while the cytoplasm is assigned to peaks at 1001 and 1660 cm^−1^, representative of phenylalanine and amide I bonds found in proteins [31,32]. Lipids are characterized by a peak at 1750 cm^−1^ explaining C=C vibrations in fatty acids [31,33]. The unknown component detected only in UCO demonstrated peaks at 1167 and 1593 cm^−1^, which might be assigned to C=N and C=C stretching in quinoid rings and C-H in plane bending [32].

### 2.2. Nuclei Features Identify Spectral Differences between Treated Organoids

**Figure 2:**
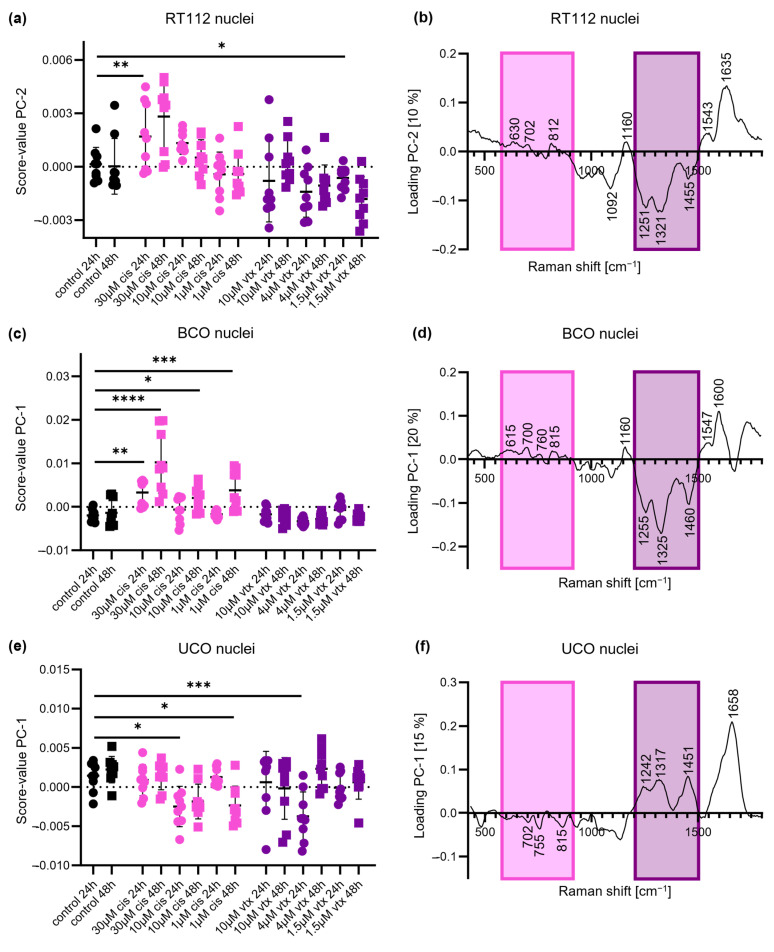
**PCA of nuclei-derived Raman spectra reveals similar spectral changes for all organoid models after *cis* (pink) and *vtx* (purple) treatments**. (**a**) Score value analysis of the cell line RT112 shows statistically significant differences after *cis* and *vtx* treatment. (**b**) Corresponding loading plot. (**c**) Score value analysis of patient-derived BCOs shows statistically significant differences after *cis* treatment and tendencies of separation after *vtx* treatment. (**d**) Corresponding loading plot. (**e**) Score value analysis of patient-derived UCOs reveals statistically significant differences between controls and *cis* and *vtx* treatment. (**f**) Corresponding loading plot. Black: controls; pink: *cis* treatment; purple: *vtx* treatment; circles: 24 h; square: 48 h; Statistical analysis: One-way ANOVA, *n* = 9, * *p* < 0.05, ** *p* < 0.01, *** *p* < 0.001, **** *p* < 0.0001.

To determine the sensitivity of RMS to evaluate drug efficacy in our organoid models, Raman spectra of nuclei were extracted from Raman images. A total number of 20 spectra per organoid and concentration with respect to the acquired timepoint were further analyzed by PCA to investigate the cellular response of the three cancer models. A comparison of PC score values demonstrated trends of separation in dependence on the selected treatment (Figure 2a,c,e). Compared to controls which exhibited score values close to 0, *cis*-treated organoids clustered at opposite score values to *vtx*-treated organoids. Overall, nuclei information was rather influenced by *cis* treatment than *vtx*. Within the group of *cis* treatment, RT112 organoids demonstrated concentration-dependent effects, whereas time-dependent trends were evident for BCO, and no clear time or concentration correlation was visualized for UCOs. 

To identify Raman peaks responsible for the separation between *vtx* and *cis* treatment, PC loadings were plotted, and prominent peaks were identified (Figure 2b,d,f). The loadings for each of the three organoid models demonstrated similar band assignments for the separation of *vtx-* and *cis*-induced effects. The shifts to positive loadings in RT112 and BCO as well as to negative loadings in UCO at 702 and 815 cm^−1^ in *cis*-treated organoids might be related to conformational changes from B-form DNA to A-form DNA [34]. Additionally, shifts at 615 and 750 cm^−1^, representative of changes in the thymidine band, are observed in *cis*-treated organoids. On the opposite, data from non-*cis*-treated organoids demonstrated more pronounced peaks at 1250, 1321, and 1455 cm^−1^, which represent guanine and DNA, respectively [30,35]. All relevant peaks and their molecular assignments are listed in Table 1.

### 2.3. Mitochondrial Spectra Identify Spectral Differences between Organoids after *vtx* Treatment

**Figure 3:**
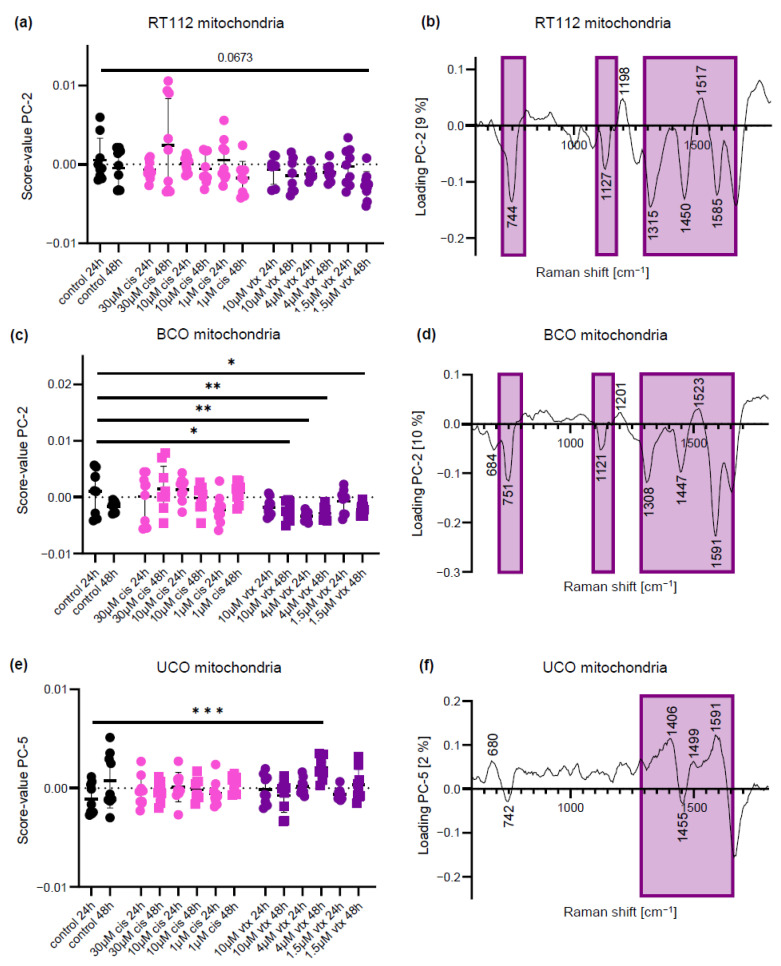
**PCA of mitochondria-derived Raman spectra reveals similar spectral for all organoid models after *vtx* (purple) treatment.** (**a**) Score value analysis of organoids from the cell line RT112 shows tendencies of separation after *vtx* treatment. (**b**) Corresponding loading plot. (**c**) Score value analysis of patient-derived BCOs show statistically significant differences after vtx. (**d**) Corresponding loading plot. (**e**) Score value analysis of patient-derived UCOs reveals statistically significant differences between controls and *vtx* treatment. (**f**) Corresponding loading plot. Black: controls; pink: *cis* treatment; purple: *vtx* treatment; circles: 24 h; square: 48 h; Statistical analysis: One-way ANOVA, *n* = 9, * *p* < 0.05, ** *p* < 0.01, *** *p* < 0.001.

PCAs were also performed on extracted mitochondria spectra from the three organoid models to assess cellular responses with regard to cell metabolism. For *cis* treatment, no significant differences in spectral signatures were observed when compared to untreated specimens in all tested organoid systems (Figure 3a,c,e). In contrast, alterations in mitochondrial spectra were observed after *vtx* treatments in patient-derived BCOs and to a smaller extent in UCOs. No significant shift was shown for cell-line-derived organoids. The corresponding loading plots (Figure 3b,d,f) exhibit, similar to the PCAs of nuclei, a recurrent pattern when comparing the individual multivariate analyses with each other. The most prominent peaks in correlation with *vtx* treatment are depicted in purple boxes in the loading plots. The peaks around 1308–1315 cm^−1^, 1125 cm^−1^ and 747 cm^−1^ can be assigned to cytochrome c [28,38]. The bands in the region between 1447–1450 cm^−1^ are indicative of changes in the CH_2_ conformation of proteins [30,31,39]. Interestingly, the cellular response to *vtx* in UCOs demonstrated inverted effects at 742 cm^−1^ and 1455 cm^−1^ when compared to the other organoid systems, presenting the biggest heterogeneity within a group and only a separation at a lower explained variance (PC-5 at 2%).

### 2.4. Data-Driven Feature Selection Identifies Novel Biomarkers

Neural networks were utilized as a complementary data-driven method to derive potential biomarkers suitable for the evaluation of spectral data and to compare feature output in dependence on the linearity of the transformation method. By using the FeaSel-Net algorithm, we aimed to find spectral biomarkers (i.e., wavenumbers) in the dataset while classifying it into one of the three classes: *cis*, *vtx* and *control*. 

In total, six different datasets (*nuclei* and *mitochondria* for each of the three organoid models) were evaluated. Each dataset initially consisted of 350 features (spectral range from 400–1800 cm^−1^ with a sampling interval of 4 cm^−1^) and was pruned to 10 features after 16 pruning iterations. During preprocessing, Raman spectra were standardized along the feature axis. Their mean spectra can be found in Figure S1. Since neural networks are inherently random in their parameter initialization, a deterministic result cannot be achieved. Thus, we statistically evaluate the resulting masks from 50 executions of the FeaSel-Net algorithm. The five most relevant wavenumbers per dataset are presented in Table 2. The corresponding percentages show how often each wavenumber has been chosen within the 50 executions. Even though the input signal was compressed to less than 3% and an equally likely selection of the features yielded approximately 1.4 selections per feature (2.85%), the algorithm found significantly increased interest in the stated features.

The most robust wavenumber eventuated in the nuclei BCO dataset with 50 selections, i.e., a selection in every run. On the other hand, the lowest percentage occurs in the UCO mitochondria dataset with 19 selections, which is still 13.5 times higher than uniformly distributed. Another interesting finding is that there are some overlaps in the resulting features, especially in the nuclei datasets. The spectral area at 1535–1555 cm^−1^ appears to be relevant to all nuclei datasets, and the area around 925–935 cm^−1^ appears to be relevant for RT112 and BCO. These possible biomarkers could be assigned to changes in DNA backbones or α-helix structure [40,41] and to changes in the amide II region [42], all denoting structural alterations of DNA. 

Another reoccurring wavenumber emerged in RT112 and BCO mitochondria around 690–705 cm^−1^. In the PCAs of mitochondria, this wavenumber was not detected in any of the loadings for separation between drug treatments and controls. The wavenumbers at around 700 cm^−1^ could be assigned to cholesterol ester and might display a reaction to drug-induced oxidative stress [36,43]. Another explanation might be the structural-altering effect of *cis* on mitochondrial DNA [33,44].

**Figure 4:**
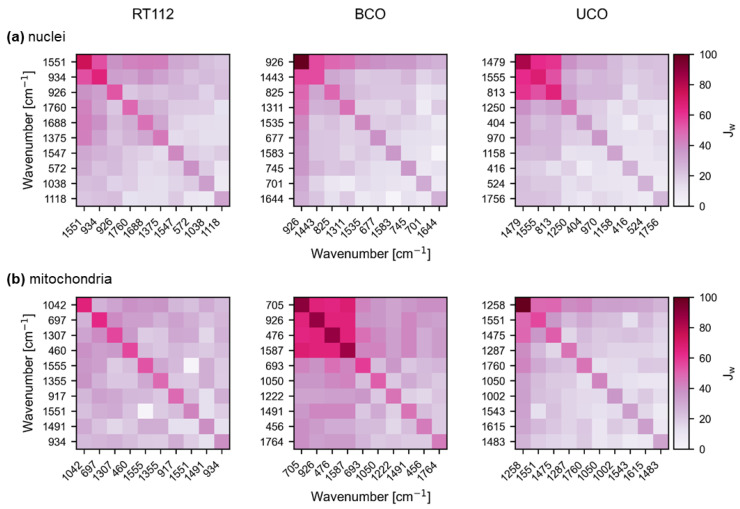
**Weighted Jaccard coefficients for the most important wavenumbers evaluated with FeaSel-Net in percent.** The plots describe the dependencies within the selected wavenumbers. Darker areas indicate a strong relationship between the features. The selection dependency for the nuclei spectra of each organoid type is shown in (**a**), whereas those of mitochondria are depicted in (**b**).

Furthermore, the interdependencies of the chosen wavenumbers were analyzed, i.e., which wavenumbers are commonly chosen in the same run. To do so, Jaccard coefficients were calculated using the masks that were obtained in 50 runs. The resulting Jaccard coefficient matrices were weighted by the number of feature occurrences in the selection. Figure 4 shows the 10 most selected wavenumbers and their dependencies for each dataset. Especially for the matrices of the BCO mitochondria and the UCO nuclei set, a clustering in the upper left corner is described, indicating a frequent selection of the most common markers at the same time. The simultaneous pick in these BCO and UCO sets cannot be perceived in the other organoid types that exhibit *XOR* behaviors rather than *AND*s, which indicates that even univariate classifications with reasonable results could be possible. In particular, the nuclei BCO set, whose most important wavenumber at 925.5 cm^−1^ is selected every run, is probably able to provide enough information on its own to separate all three classes correctly. 

### 2.5. Classification with Selected Raman Markers

The performance of the biomarkers retrieved from the FeaSel-Net algorithm (Table 2) and from conventional loading analysis of the PCAs (Table S1) was analyzed in the following. Accordingly, the original Raman features were masked and reduced for each dataset and used for classification. The applied classifier for both PCA-derived and FeaSel-Net derived methods was the same fully-connected neural network with an input of five discrete wavenumber values (masked original Raman signal) and an output of the three classes *vtx*, *cis* and *control*. We purposely did not use the classification model from our FeaSel-Net algorithm to ensure equal chances for the two different feature selection methods. 

Table 3 shows the classification results with the performance parameters’ accuracy (ACC), sensitivity (SEN), and specificity (SPE) for the neural network trained with the masked RMS data for each of the six datasets. The neural network was trained 10 times for each dataset and feature selection. At first glance, striking classification accuracies between 73 and 87% were demonstrated when taking into account that less than 1% of the overall spectral data points were utilized for the discrimination. In both organelles, the cell line and the BCO model performed slightly better than the UCO. 

When comparing the two biomarker selection methods, Table 3 indicates a difference between the conventional feature selection method (PCA loadings) and the data-driven approach (FeaSel-Net). Throughout all datasets, classification parameters were improved. The overall accuracy increased by 7.3%, whereas improvement in specificity and sensitivity accounted for 5.1% and 12.8%, respectively. These effects correlate in particular to the nuclei-based classification between *control*, *vtx* and *cis* (Table S2 provides a detailed overview).

**Figure 5:**
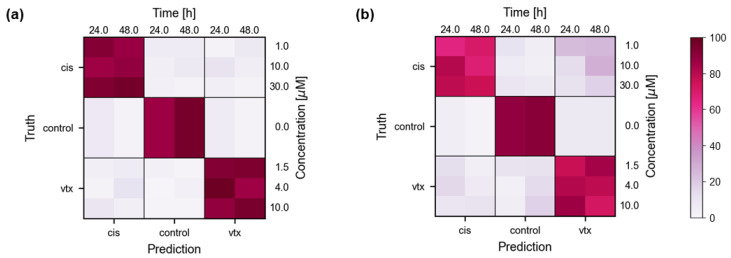
**Confusion matrices for the BCO nuclei dataset resolved by drug concentration and exposure time.** Classification accuracy (%) with features retrieved from FeaSel-Net (**a**) or PCA loadings (**b**) are shown.

The previous analysis did not consider differences emerging from the drug concentrations or treatment durations. Thus, classification was repeated on the best-performing pre-trained model (BCO nuclei), and the discrimination was additionally split into concentration and exposure time-dependent subsets. The resulting confusion matrices for different input features are shown in Figure 5. Contrary to the assumption that longer exposure times and higher doses yield better discriminability, a trend that confirms these assumptions cannot be described. This could be since the training has been done with data from all concentrations and durations. However, even though Figure 5 does not show any correlations between drug doses and exposure time, it still shows the influence of the selected biomarker features. The classifier with an input defined by FeaSel-Net (Figure 5a) performs better than a classification with the PCA-derived wavenumbers (Figure 5b). 

### 2.6. FLIM Enables Non-Invasive Monitoring of Drug Response Patterns in Bladder Cancer Organoids

**Figure 6:**
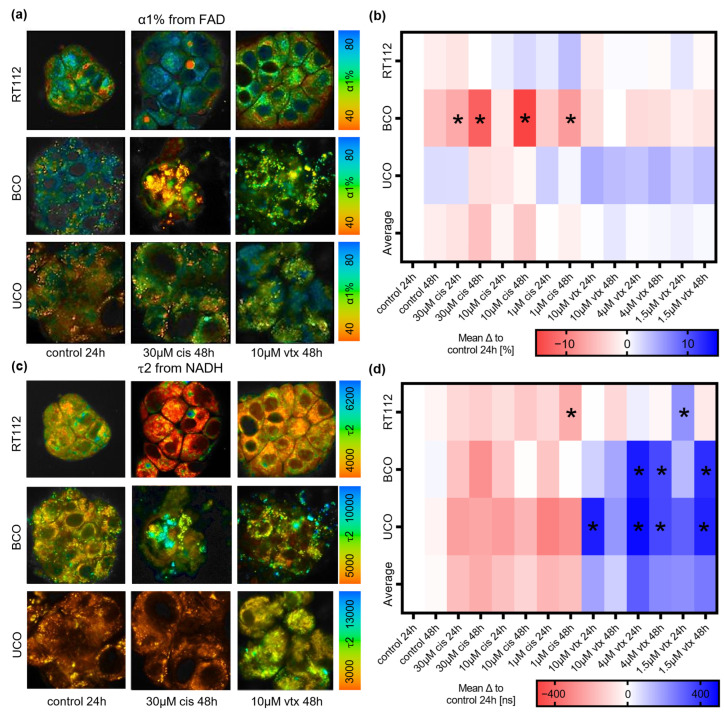
**FLIM of bladder cancer organoids.** (**a**) Representative FAD α1% images of control RT112, BCO and UCO organoids and 48 h after 30 µM *cis* or 10 µM *vtx* treatment. Scale bar: 25 µm. (**b**) Heatmap of mean differences of NADH α1% to the 24 h control. (**c**) Representative NADH τ2 images of control RT112, BCO and UCO organoids and 48 h after 30 µM *cis* and 10 µM *vtx* treatment. Scale bar: 25 µm. (**d**) Heatmap of mean differences of NADH τ2 to the 24 h control. One-way ANOVA, *n* = 15, * *p* < 0.05.

Drug treatment with *cis* or *vtx* influences the metabolic pathways in cells [45,46], and it has been shown that FLIM identifies metabolic changes in cell cultures and in vivo [47]. Therefore, FLIM images of endogenous NADH and FAD fluorescence were acquired from RT112, BCOs, and UCOs treated with different concentrations of *cis* and *vtx* for 24 and 48 h. For each treatment, FLIM parameters τ1, τ2, and α1% of the respective coenzymes NADH and FAD were characterized and compared among each other. Exemplarily, differences in FAD α1% and bound NADH fluorescence lifetime τ2 are visualized in representative FLIM images (Figure 6a,c) and mean difference heatmaps (Figure 6b,d). The complete overview of NADH and FAD τ1, τ2, and α1% values can be found in the supplementary material (Figures S2–S4). 

Comparing NADH and FAD a1% readouts, representing the redistribution of free vs. bound coenzymes, no significant changes were observed upon *vtx* treatment for any of the organoid models. On the contrary, upon *cis* treatment, a decrease in a1% in both NADH and FAD was observed for BCOs (Figure S2b,e) as well as in FAD a1% in UCOs (Figure 6b). Cell line-derived organoids did not indicate changes in a1% (Figure S2a,d). 

FLIM parameters representing the fluorescence lifetime of free (τ1) and bound (τ2) NADH and free (τ2) and bound (τ1) FAD exhibited statistically significant changes for all organoid models after *vtx* treatment and a partial impact of *cis* treatment. Lifetimes τ1 and τ2 of both NADH and FAD increased after *vtx* treatment in all organoid models (Figure S2). In addition, an increase in NADH τ1 after treatment with a high concentration of *cis* was observed in the RT112 and BCO models after 48 h (Figure S3d). In UCO, *cis* lead to a significant decrease in FAD τ1 (Figure S4b). Overall, except for NADH τ1, mean difference heatmaps indicate opposite effects on fluorescence lifetime upon *vtx* and *cis* treatments.

### 2.7. Feature Importance Analysis Automatically Identifies Most Informative FLIM Parameters

Conventional statistical comparison, i.e., via ANOVA, requires manual interpretation and comparison of each FLIM parameter and is highly dependent on sample heterogeneity or standard deviation. Thus, we were interested in investigating whether a subset of FLIM parameters existed that was sufficiently informative of changes resulting from different drug treatments. To assess the ability of FLIM parameters to automatically discriminate organoids treated with *cis* versus *vtx*, we conducted a feature importance clustering analysis. For this analysis, we considered averaged values of FLIM parameters over all biological replicas or each timepoint/drug/model. We excluded control measurements from this analysis because our focus was on differentiating cells treated with *cis* from those treated with *vtx.* We first verified that FLIM parameter values could be used to automatically separate *cis*-treated organoids from *vtx*-treated ones for each model. Using a k-means clustering analysis (with k = 2), we were able to correctly divide each dataset into two clusters, one containing only *cis*-associated values and the other containing only *vtx*-associated values. These results were consistent across all three models (RT112, BCO and UCO). We then sought to identify which parameters were driving the cluster assignment. To assess the relative importance of each parameter, we conducted a feature importance study. We were able to identify a lower-dimensional subspace of features that offered the correct separation of our datasets. This lower-dimensional subspace contained two out of the six FLIM parameters which were found to be sufficiently informative to discriminate *cis*-treated organoids from *vtx*-treated organoids. Interestingly, the most informative pairs differed across models (Table 4). These results suggest some general trends. First, fluorescence lifetimes seemed to be most informative for cell line and patient-derived models, although different channels were selected for each model. Second, the fluorescence lifetime of τ2 from NADH is the only parameter selected in more than one model, indicating resemblances in patient-derived organoids compared to the cell line. 

## 3. Discussion

In this study, RMS and FLIM were utilized for a comprehensive characterization of the drug effect of *cis* and *vtx* on bladder cancer organoids in different models derived from a cell line as well as patient-derived primary tissue and urine. Our RMS and FLIM measurements suggest that the spectral and endogenous fluorescence information, especially from mitochondria and nuclei, can be useful in situ tools for non-destructive monitoring of drug effects on organoid models. 

The promise of patient-derived organoid models in precision medicine relies upon the notion that characterization of their mutational profiles in combination with high-throughput screening with a library of therapeutic compounds can elucidate druggable targets. In the case of bladder cancer, patients are often diagnosed early in disease progression, and patients with non-muscle invasive cancer frequently undergo multiple resections and treatments to avoid cystectomy and its detrimental impact on quality of life. Thus, information on effective drug candidates identified by screening in personalized organoid culture could be utilized to guide intravesical therapies and support decision making for earlier therapy success and better patient compliance by avoiding side effects. In recent years, RMS and FLIM have been established as promising techniques for investigating molecular and metabolic changes in cells. The advantages of these methods are their non-destructive approach with concomitant spatial resolution at the subcellular level [3,23,48].

Therefore, we investigated the capability of these techniques to monitor the metabolic and molecular response of bladder cancer organoids to chemotherapeutic drugs at spatial and temporal resolution. TCA-based image generation enabled the marker-independent discrimination and localization of major subcellular structures within the organoids and even allowed the visualization of accumulations of the drugs. Intensity distribution heatmaps of the single components enabled us to further investigate underlying spectral information. Spectral signatures provided access to changes in molecular composition and identified drug-specific peak patterns in nuclei-derived PCA loadings, which reoccurred in each of the three organoid models. *Cis* treatment-related changes were correlated to alterations in the structure of DNA. Pronounced peaks at 700 and 815 cm^−1^ reported in A-form DNA [34] were present in all organoid models. Multiple studies demonstrate that *cis* results in intrastrand crosslinks (CLs) between adjacent purine bases (1,2-GG or 1,2-AG CLs) or between purine bases separated by a third base, CLs, and monofunctional adducts [49,50]. Additional shifts were observed at 615 and 750 cm^−1^, which identify C2′-endo/anti conformers of deoxy thymine [34,37] and support our conclusion that RMS has the fidelity to screen for variations in DNA structure. 

In addition to DNA damage, *cis* is also known to induce the production of ROS in its target cell [51,52,53]. Although the intracellular origin of ROS production is still unclear, it was reported that especially *cis*-induced ROS production occurs in mitochondria [54]. Generation of ROS often correlates to loss of the mitochondrial membrane potential [55], leading to inhibition of the TCA cycle [51], which can impact fluorescence lifetimes and is, therefore, a potential reason for alteration upon *cis* treatment. NADH τ1 denotes the fluorescence lifetime of free, cytosolic NADH, in contrast to bound NADH, which is found in the oxidative phosphorylation chain and is designated by τ2 [56]. Fluorescent lifetimes are highly sensitive to alterations in the cellular microenvironment, such as pH, solvent polarity, or even viscosity and can, therefore, directly monitor changes in organoids [47,57]. Our data of *cis*-treated organoids indicate similar trends among τ1 and τ2 for both NADH and FAD but were only significant for 30 µM *cis* concentration. 

In contrast, *vtx* treatment significantly affected both FLIM and Raman results for all organoid models. *Vtx* is a chemotherapeutic drug mimicking the BH3 domain of pro-apoptotic proteins capable of binding to and antagonizing BCL-2 family anti-apoptotic proteins. As a result, the cell undergoes apoptosis mediated by the mitochondrial pathway and initiated by the activation of caspases [58]. This effect was reflected in the Raman data of mitochondria, demonstrating shifts in wavenumbers relevant to cytochrome c. The latter were found mainly in BCOs and UCOs. Utilizing PCAs, nuclei-related alterations were also observed upon *vtx* treatment, which, unlike *cis*, does not directly interact with DNA. We assume that the recurrent changes in the loading plots at 1255, 1325, and 1455 cm^−1^, all representative of DNA, especially guanine, correlate with the preparation for apoptosis or altered cell metabolism. These shifts might refer to the concomitant denser packing of cell nuclei during the initiation of apoptosis [59]. 

FLIM was able to detect statistically significant effects on NADH and FAD lifetimes for all three organoid models after *vtx* treatment. In all models, we found increased fluorescence lifetimes as a reaction of cells to *vtx* probably undergoing apoptosis. These findings are consistent with the results of other studies reporting an increase in NADH lifetime associated with apoptosis [60]. 

In comparison to significant changes in fluorescence lifetimes, minor drug-induced effects were reflected in NADH and FAD α1% values and only detected for BCOs treated with *cis*. A decrease in NADH α1% is associated with a shift in the ratio of free to bound NADH, which correlates to a switch from glycolysis, favored in cancer cells, to oxidative phosphorylation. Another explanation for the changes in α1% might be an increased energy consumption of nuclei as they prepare for apoptosis [61,62], i.e., after DNA bending due to the impact of *cis*. 

However, when comparing NADH and FAD α1% among all organoid systems, differences in control organoid baseline values were observed. While NADH a1% was at ~78% in non-treated RT112, decreased baseline values were observed in BCOs and UCOs between 60–80% and 50–70%, respectively. These baseline shifts were also visible for α1% of FAD to decreased values in BCO and UCO compared to RT112. An explanation for this result might be the stage and type of cancer, which is reflected by the different models. The cell line RT112 is established from a G2 transitional cell carcinoma with untreated primary urinary bladder carcinoma. BCOs were retrieved from a muscle-invasive bladder cancer tumor in stage pT2 in G3, while UCOs were derived from a less aggressive surface tumor in stage pT1 in G2. Because α1% is a direct measure of metabolic state indicating the balance between glycolysis and oxidative phosphorylation, baseline shifts in non-treated organoids were in accordance with the metabolic activity and severity of the cancer state.

In addition to the introduction of non-destructive readouts for analyzing the cytotoxic effects of drugs on different types of organoids, we aimed to build data-driven classification models that allow us to identify the most robust and relevant Raman and FLIM parameters enabling the identification of novel biomarkers. Both FLIM and RMS measurements generate large data sets, and automated dimensionality reduction and feature selection can help to translate these methods into a clinical setting and improve the interpretability of the data. The establishment of neural networks allowed us to identify different Raman markers relevant for the separation between controls and treated organoids across the organoid systems. With non-linear optimization and transformation processes that are inherent to neural networks and the recursive feature evaluation provided by FeaSel-Net, the extraction of the most relevant data subsets was enabled in a more complex and yet more accessible approach. The advantage of non-linearity has been confirmed in a deep-learning-based classification with the elaborated features. With Jaccard matrices, the interdependence of the features and the need to apply multivariate analyses on spectral data have been shown. Retrieval of Raman signals from data-driven feature selection results in 12.2% improved sensitivity for discrimination of nuclei-related effects and 6.8% improved sensitivity for drug-induced mitochondrial changes in comparison to Raman bands guided by PCA loadings. 

It must be stated that the data-driven and single organoid-based approach presented in this study is very sample-specific, and its finding must not be assumed for bladder cancer in general. To further evaluate the robustness of this method for the assessment of drug response and, in particular, to improve sensitivity to dose-dependent effects, our experiments would need to be repeated on multiple donors with parallel viability assays. Another factor to be considered for this data analysis approach is its virtue: the non-linearity. Compared to PCAs and their assigned loadings plot, very deep neural networks can find patterns in the signal that are not interpretable by humans or statistical analyses. We have specifically chosen the FeaSel-Net approach for feature selection since it offers more possibilities to fine-tune hyper-parameters, which enables us to find better results. Another reason for the neural network approach, in contrast to other feature selection methods such as XGBoost [63], is the capability of further optimizing the machine learning model by adapting the pre-trained weights from our previous feature selection. In terms of generalizability, the neural network approach will perform better when having a bigger dataset and multiple outliers or noisy data.

FLIM measurements obtain multiple parameters of bound and free FAD and NADH, characterizing the metabolic profile of cells. To perform faster and more robust analyses of FLIM data, we investigated whether the identification of different drug effects is reflected in a smaller subset of parameters. All organoid models were subjected to data-driven k-means clustering to identify the FLIM parameters explaining the drug-induced effects. Among organoid models, only one parameter overlap in τ2 NADH was detected for BCO and UCO organoids. We hypothesize that this indicates similarities in metabolic activities between the patient-derived models but differences in the cell line. Since the RT112 cell line exhibits no overlap in relevant FLIM parameters to the patient-derived organoids, the metabolic and biological difference between the models becomes evident, highlighting the need for patient-specific analyses in drug testing. The overlap of the selected FLIM parameters further corroborates the comparability of the organoid systems, allowing urine-derived organoids to be considered equivalent to organoids derived from primary tissue despite their origin from different donors. K-means clustering also highlights that FLIM parameters displaying the ratios of bound to free NADH and FAD are less important to discriminate the drug effects between *cis* and *vtx* as only in UCO was α1% of FAD selected. One possible reason for this result could be that the two drugs tested may have a relatively smaller effect on the redistribution of NADH and FAD than on the change in the microenvironment of the cell, which is detected very sensitively by alterations in the fluorescence lifetimes. 

With the novel data-driven methodologies presented in this study, we provide first insights into similar molecular behaviors upon drug treatments between patient-derived organoids produced from primary tissues compared to urine. Interestingly, we found variabilities between the patient-derived organoids and the cell line, especially by clustering the FLIM parameters and thus highlighting the need for patient-specific analysis. These evaluation methods give insights into the distinctness of treated and control organoids, but also provide information on the importance of the specific features of the data, and subsequent filtering allows us to focus on the relevant data. The purpose of the extraction and evaluation of relevant features is to enhance generalizability during discrimination tasks, therefore relieving the process of sample acquisition and improving discriminability. With this knowledge, new sparse sensors and measurement protocols can avail faster and more efficient tumor classifications up to real-time evaluations. This methodology could also be applicable in the future as a tool to identify biomarkers that can be utilized to distinguish tumorous from healthy cells. An important future direction is to conduct Raman and FLIM experiments on additional patient-derived organoids to investigate donor-specific variances. The comparisons between urine- and tissue-derived organoids from the same patient would be of particular interest for further evaluation of both methods. An additional target for subsequent studies is the comparison with patient-derived organoids retrieved from normal bladder tissue. This will allow the identification of the most effective drug concentrations that have a maximum effect on the cancerous tissue and minimum side effects.

The ultimate goal of our study was to establish a multiparametric workflow to rapidly evaluate drug-induced effects on patient-derived tissue models. With our experiments, we provide evidence that RMS and FLIM on organoids can be utilized as a test platform to evaluate the effectiveness of different anticancer drugs as well as their mode of action. In addition, we demonstrated that data-driven approaches can be utilized to reduce data complexity by automated feature selection enabling to improve of classification models and ultimately lead to better prediction accuracy. 

## 4. Materials and Methods

### 4.1. Cell Culture of Organoids in Matrigel

After informed consent of the patient, tumor cells were retrieved from surgical specimens of a radical cystectomy or rinsing urine for BCO and UCO, respectively. The study was approved by the Ethics Committee (804/2020/B02). An overview of patient-specific pathohistological information is given in Table S3. For the preparation of BCO, the tissue was covered by working medium (DMEM, 2 mM glutamine, 10% FBS, 1% pen-strep (all from Sigma-Aldrich, St. Louis, MO, USA), 100 mM Y-27632 (MecChemExpress, Hölzl GmbH, Cologne, Germany), mechanically cut in cubes of approximately 1 mm^3^, suspended in 10 mL working medium and centrifuged (480 g, 10 min, ambient temperature). The sediment was resuspended in 1 mL PBS per 100 mg tissue wet weight. Extracellular matrix components were enzymatically degraded through the addition of 15 µL of a blend of collagenase (3000 U/mL)/hyaluronidase (1000 U/mL; STEMCELL Technologies, Vancouver, Canada) per 1 mL suspension and incubated for 30 min at 37 °C. This step was repeated once. Subsequently, undissolved tissue was removed by a 70 µm cell strainer, and the filtrate was centrifuged (150 g, 7 min, ambient temperature). The vital cells were counted and resuspended at 1 × 10^6^ cells/mL in organoid culture medium and cooled on wet ice [9,64]. The cell suspension (30 µL) and Matrigel (10 µL, BioTechne, Minneapolis, MN, USA) were mixed on ice. 

This blend of cells in Matrigel (40 µL) was placed in a 24-well plate. The plate was then turned upside down and incubated for 5 min at 37 °C to generate hanging drops. A total of 500 μL organoid culture medium [9,64] was added per well and incubated at 37 °C, 5% CO_2_ and a humidified atmosphere. The organoid culture medium was routinely replaced twice a week after microscopic evaluation of cell growth of the organoids. Before measurements, organoids were transferred into 8 well μ-slides (ibidi GmbH, Gräfelfing, Germany). For the preparation of UCO, cells from urine samples were sedimented by centrifugation, washed twice with PBS, counted, and resuspended at 1 × 10^6^ cells/mL in organoid medium to generate organoids as described above.

### 4.2. Immunofluorescence Staining

For imaging, organoids were cultured in 8-well chamber slides. Organoids were fixed by 4% formaldehyde (30 min, ambient temperature) and blocked (5% BSA, 0.2% Triton X-100, 0.1% Tween 20, in PBS; 1 h, ambient temperature), and incubated (1 h, 37 °C) with primary antibodies CK5 (BioLegend, Amsterdam, The Netherlands), CK 7 (Abcam, Cambridge, UK) and GATA3 (Abcam). Primary antibodies were incubated with complementary Alexa Fluor 488-labelled (Jackson ImmoResearch Europe, Cambridge, UK) secondary antibodies (1:250, 1 h, ambient temperature). Nuclei were counterstained by DAPI (DAKO), and the expression of the marker genes was visualized by microscopy (Zeiss Axiophot, Carl Zeiss AG, Oberkochen, Germany). Antibody diluent was 1% BSA in PBS. Samples without primary antibodies and samples stained with anti-rabbit IgG antibodies served as controls. 

### 4.3. Sample Preparation for Spectroscopic Raman- and FLIM Measurements

Organoids derived from the RT112 cell line, primary tumor tissue and urine were incubated with 10 µM, 4 µM and 1.5 µM venetoclax (*vtx*, Sellek Chemicals, Houston, TX, USA) or 30 µM, 10 µM and 1 µM cisplatin (*cis*, Sellek Chemicals) in cell culture medium for 24–48 h at 37 °C in 5% CO_2_ atmosphere. *Vtx* was dissolved in 20% Captisol (Sellek Chemicals), while *cis* was dissolved in Milli-Q water. Controls were kept in medium. Organoids were first measured with FLIM and afterward with RMS. Prior to FLIM measurements, the samples were washed with PBS to remove phenol red interfering with Raman measurements. Organoids were kept in 200 µL PBS throughout the measurements and immersed in a cell culture medium with or without drugs afterward. 

### 4.4. FLIM Imaging of Organoids

Time-correlated single-photon counting (TCSPC) fluorescence decay measurements were performed with a Zeiss LSM 880 (Zeiss) coupled with a Ti:Sapphire femtosecond laser (MaiTai HP Spectra Physics, Santa Clara, CA, USA) and a two-channel NDD BIG2.0 GaAsP PMT detector (Becker & Hickl GmbH, Berlin, Germany). Autofluorescence of NADH and FAD was excited with two-photon excitation at a wavelength of 700 nm and 5% laser power through a 63×1.4 NA C-plan apochromat objective (Zeiss). Emission light was filtered in the range of 450 to 490 nm for NADH and 500 to 550 nm for FAD. The total image acquisition time was set to 141 s at a resolution of 512 × 512 pixels and a pixel dwell time of 32.77 µs. The instrument response function was recorded at 900 nm from crystalline urea (Sigma-Aldrich). All FLIM measurements were performed at 37 °C using a microscope stage top incubation system (ibidi heating system, ibidi GmbH).

### 4.5. FLIM Data Analysis

SPCImage (Becker & Hickl GmbH) was utilized to perform biexponential decay fittings with a 30% threshold of maximum photon count to remove the background. The quality of fit was decided based on a mean χ^2^ value smaller than 1.15 per image. ASCII images for α1%, τ1 and τ2 were exported for both NADH and FAD for further analysis. α1% explains the ratio of bound to unbound FAD or the ratio of unbound to bound NADH and is a direct measure of cell metabolism. The fluorescence lifetimes τ1 and τ2 describe the fast and slow components of exponential decay.

### 4.6. Raman Imaging of Organoids

Spectral Raman mapping was performed on a customized inverted WITec alpha300 R Raman system (WITec GmbH, Ulm, Germany) equipped with a green laser (532 nm) and a CCD spectrograph with a grating of 600 g/mm. An incubation chamber (Okolab S.R.L.) was integrated into the setup to keep the organoids constantly at 37 °C. Images were acquired from at least three organoids at a laser power of 58 mW, an integration time per spectrum of 0.2 s and a pixel resolution of 1 × 1 μm at a size of 50 × 50 µm. All measurements were acquired with a 50× objective (Carl Zeiss AG, Oberkochen, Germany). Reference spectra of cytochrome c (derived from bovine heart, Sigma Aldrich), *cis* and *vtx* were recorded as single spectra with an accumulation of 10 spectra and 0.5 s integration time.

### 4.7. Multivariate Data Analysis

Image analysis of spectral maps was performed with the Project Five 5.2 software (WITec GmbH, Ulm, Germany). RMS data were preprocessed in regard to cosmic ray removal, polynomial baseline correction, cropping to 400–3000 cm^−1^ as well as area intensity normalization. True component analysis (TCA) was performed to analyze Raman images. In brief, TCA is a non-negative matrix factorization-based multivariate data analysis tool elaborating spectral components, which predominantly occur in the data set, allowing us to identify their spectral distribution by false color intensity distribution heatmaps. Based on TCA heatmaps, spectral information (20 spectra/organoid) representing nuclei or mitochondria was extracted for further in-depth analysis of the molecular composition by principal component analysis (PCA) using Unscrambler X10.5 (Camo, Norway). PCA is a gold standard multivariate data analysis tool for spectroscopic data, reducing the dimensionality of a set of spectral data on a vector-based approach. Each vector, the so-called principal component (PC), describes a variation in the spectra. Plotting PC values against each other visualizes a correlation or separation of two or more data sets. The interpretation of the underlying spectral changes can be derived from the PC loadings plot. 

### 4.8. Feature Selection Using FeaSel-Net

We recently developed the neural network architecture FeaSel-Net that is capable of recursively selecting relevant wavenumber areas (features) in the classifier’s input signal [65]. It is a combination of a neural network classifier and a feature selection algorithm. Other than dropout methods, this approach does not focus on stochastic pruning of parameters within hidden layers to improve generalizability but on deterministically pruning nodes in the input layer [66]. The package is open-source and can be downloaded from https://pypi.org/project/FeaSel-Net/, (Version 0.0.1). When features are selected, the optimizer in the neural network has to adapt to the fewer input signals and re-optimize the classifier with the new requirements. The selection process itself is a rather complex procedure, where the entropy is measured for every feature being masked, and the features with the highest entropy are kept. Contrarily to the interpretation of PCA loadings, this method provides a completely data-driven and unbiased evaluation of the findings and serves as an extension of the former method. In our recursive feature selection process, every selection was made whenever the threshold of classification accuracy of τ_acc_ = 0.98 was consistently surpassed in the optimization process. The features were reduced by 20% every time the feature evaluation was executed. The reduction was made by masking the initial signal at the input of the neural network. All other parameters can be retrieved from Table S4.

### 4.9. Neural Network Classifier

Sample discrimination was undertaken by using a simple neural network from the open-source Keras and Tensorflow API (Google Brain). The model had a rhomboidal fully-connected layer structure with 5−10−20−10−3 nodes, where the layer with 5 nodes was the input layer, and the one with 3 nodes was the output layer. The activation functions used were mainly ReLU (Rectified Linear Unit) and one sigmoid function in the last layer for probabilistic output values. The optimizer used was Adam [67], with a learning rate of η=0.005. With a batch size of 128, the model was trained for 100 epochs, and a train test split of 0.8 was applied. The model was tested with all validation and training data to obtain the performance parameters in Section 0.

### 4.10. Feature Importance Clustering Analysis

In total, we curated three model-specific datasets (RT112, BCO and UCO), each with 72 measurements (six FLIM parameter values across 12 timepoints/drug concentration). We first scaled all data to the z-norm. This was done to ensure that the clustering algorithm can focus on structural similarities and differences instead of amplitude-driven ones. We then performed clustering analysis using the method implemented in the R package *kmeans* for k = 2. Next, we performed a feature importance analysis using the function *FeatureImpCluster* in R by setting the number of true clusters to 2. We repeated the feature importance analysis 20 times using different random seeds. The function *FeatureImpCluster* computes the permutation misclassification rate for each variable of the data. The mean misclassification rate over all iterations can be interpreted as variable importance.

### 4.11. Statistical Analysis

Statistical analysis was performed using GraphPad Prism version 9.00 for Windows (GraphPad Software). Results are shown throughout the entire article as mean ± standard deviation. All data sets are tested for normal distribution using the Kolmogorov–Smirnov test; outliers were removed using Grubb’s test with a confidence interval of 0.05. All n-numbers, applied tests, and corresponding significance for each result are listed in the figure legends. Experiments were performed at least 3 times.

## Figures and Tables

**Table 1:** Biological assignment of the most relevant wavenumbers.

Wavenumber [cm^−1^]	Biological Origin	Literature
702	A-form DNA	[34]
705	Cholesterol ester	[36]
747	Cytochrome c	[28]
798	PO_2_^−^	[30]
815	A-form DNA	[34]
1096	PO_2_^−^	[30,37]
1125	Cytochrome c	[28]
1250	G	[35]
1315	Cytochrome c	[38]
1321	G	[30,35]
1450	CH_2_	[30,31,39]
1455	DNA	[35]
1660	Amide I	[32]
1750	C=C	[33]

**Table 2:** Most frequently occurring wavenumbers upon treatment discrimination.

Dataset	*ν*_1_ [cm^−1^]	*ν*_2_ [cm^−1^]	*ν*_3_ [cm^−1^]	*ν*_4_ [cm^−1^]	*ν*_5_ [cm^−1^]
*Nuclei*					
RT112	1551 (76%)	934 (68%)	926 (56%)	1760 (52%)	1688 (50%)
BCO	926 (100%)	1443 (58%)	825 (52%)	1311 (50%)	1535 (44%)
UCO	1479 (84%)	1555 (70%)	813 (68%)	1250 (48%)	404 (40%)
*Mitochondria*					
RT112	1042 (54%)	697 (52%)	1307 (46%)	460 (46%)	1555 (44%)
BCO	705 (74%)	926 (72%)	476 (72%)	1587 (70%)	693 (48%)
UCO	1259 (82%)	1551 (46%)	1475 (42%)	1287 (38%)	1760 (38%)

**Table 3:** Classification performance of masked Raman data. The presented values (±SD) are the parameters’ percentage averages of 10 training runs for each dataset and feature selection method.

	PCA Loadings			FeaSel-Net		
Dataset	ACC	SEN	SPE	ACC	SEN	SPE
*Nuclei*						
RT112	76.6 ± 1.0	64.9 ± 3.9	82.4 ± 2.4	87.0 ± 1.0	80.4 ± 2.6	90.2 ± 1.3
BCO	79.0 ± 1.1	68.5 ± 3.7	84.2 ± 2.3	84.7 ± 0.8	77.1 ± 2.9	88.5 ± 1.4
UCO	73.1 ± 1.9	59.6 ± 6.4	79.8 ± 4.8	80.1 ± 1.0	70.1 ± 2.8	85.1 ± 1.6
*Mitochondria*						
RT112	77.9 ± 1.4	66.9 ± 4.6	83.5 ± 3.0	84.4 ± 0.9	76.7 ± 2.9	88.3 ± 1.8
BCO	83.1 ± 0.5	74.6 ± 1.5	87.3 ± 1.0	84.7 ± 0.6	77.0 ± 3.2	88.5 ± 2.0
UCO	72.8 ± 1.0	59.3 ± 3.7	79.6 ± 1.9	76.8 ± 1.2	65.3 ± 4.2	82.6 ± 2.6

**Table 4:** FLIM parameters selected using a feature importance analysis.

Dataset		
RT112	τ1 from FAD	τ1 from NADH
BCO	τ2 from FAD	τ2 from NADH
UCO	α1% of FAD	τ2 from NADH

## Data Availability

All data are contained within the manuscript or supplementary material. Raw data are available on reasonable request from the corresponding author.

## Supplementary Materials

The following supporting information can be downloaded at: https://www.mdpi.com/article/10.3390/ijms23136956/s1.

## References

[1] Moreira L., Bakir B., Chatterji P., Dantes Z., Reichert M., Rustgi A.K. (2018). Pancreas 3D Organoids: Current and Future Aspects as a Research Platform for Personalized Medicine in Pancreatic Cancer. Cell. Mol. Gastroenterol. Hepatol..

[2] Walsh A.J., Castellanos J.A., Nagathihalli N.S., Merchant N.B., Skala M.C. (2016). Induced Metabolism Changes in Murine and Human Pancreatic Cancer Organoids Reveals Heterogeneous Drug Response. Pancreas.

[3] Sharick J.T., Walsh C.M., Sprackling C.M., Pasch C.A., Pham D.L., Esbona K., Choudhary A., Garcia-Valera R., Burkard M.E., McGregor S.M. (2020). Metabolic Heterogeneity in Patient Tumor-Derived Organoids by Primary Site and Drug Treatment. Front. Oncol..

[4] Lin V.J.T., Hu J., Zolekar A., Yan L.-J., Wang Y.-C. (2020). Urine Sample-Derived Cerebral Organoids Suitable for Studying Neurodevelopment and Pharmacological Responses. Front. Cell Dev. Biol..

[5] Boj S.F., Hwang C.I., Baker L.A., Chio I.I.C., Engle D.D., Corbo V., Jager M., Ponz-Sarvise M., Tiriac H., Spector M.S. (2015). Organoid Models of Human and Mouse Ductal Pancreatic Cancer. Cell.

[6] Earl J., Rico D., Carrillo-De-Santa-Pau E., Rodríguez-Santiago B., Méndez-Pertuz M., Auer H., Gómez G., Grossman H.B., Pisano D.G., Schulz W.A. (2015). The UBC-40 Urothelial Bladder Cancer cell line index: A genomic resource for functional studies. BMC Genom..

[7] Kobayashi T., Owczarek T.B., McKiernan J.M., Abate-Shen C. (2015). Modelling bladder cancer in mice: Opportunities and challenges. Nat. Rev. Cancer.

[8] Lee S.H., Hu W., Matulay J.T., Silva M.V., Owczarek T.B., Kim K., Chua C.W., Barlow L.J., Kandoth C., Williams A.B. (2018). Tumor Evolution and Drug Response in Patient-Derived Organoid Models of Bladder Cancer. Cell.

[9] Mullenders J., De Jongh E., Brousali A., Roosen M., Blom J.P.A., Begthel H., Korving J., Jonges T., Kranenburg O., Meijer R. (2019). Mouse and human urothelial cancer organoids: A tool for bladder cancer research. Proc. Natl. Acad. Sci. USA.

[10] Pollehne P., Mojarro I.A.M., Schneider J., Amend B., Fend F., Stenzl A., Aicher W.K., Harland N. (2022). MP06-06 Establishment and Evaluation of a simplified approach to patient derived bladder cancer organoids using urine. J. Urol..

[11] Chin L., Andersen J.N., Futreal P.A. (2011). Cancer genomics: From discovery science to personalized medicine. Nat. Med..

[12] Diamandis M., White N.M.A., Yousef G.M. (2010). Personalized Medicine: Marking a New Epoch in Cancer Patient Management. Mol. Cancer Res..

[13] Fenstermacher D.A., Wenham R.M., Rollison D.E., Dalton W.S. (2011). Implementing Personalized Medicine in a Cancer Center. Cancer J..

[14] Marusyk A., Polyak K. (2010). Tumor heterogeneity: Causes and consequences. Biochim. Biophys. Acta (BBA) Rev. Cancer.

[15] Junttila M.R., De Sauvage F.J. (2013). Influence of tumour micro-environment heterogeneity on therapeutic response. Nature.

[16] Gong X., Lin C., Cheng J., Su J., Zhao H., Liu T., Wen X., Zhao P. (2015). Generation of Multicellular Tumor Spheroids with Microwell-Based Agarose Scaffolds for Drug Testing. PLoS ONE.

[17] Heikal A.A. (2010). Intracellular coenzymes as natural biomarkers for metabolic activities and mitochondrial anomalies. Biomark. Med..

[18] Warburg O., Wind F., Negelein E. (1927). The metabolism of tumors in the body. J. Gen. Physiol..

[19] Pettit F.H., Pelley J.W., Reed L.J. (1975). Regulation of pyruvate dehydrogenase kinase and phosphatase by acetyl-CoA/CoA and NADH/NAD ratios. Biochem. Biophys. Res. Commun..

[20] Fong E.J., Strelez C., Mumenthaler S.M. (2020). A Perspective on Expanding Our Understanding of Cancer Treatments by Integrating Approaches from the Biological and Physical Sciences. SLAS Discov..

[21] Niaura G. (2014). Raman Spectroscopy in Analysis of Biomolecules. Encyclopedia of Analytical Chemistry: Applications, Theory and Instrumentation.

[22] Chen P., Zhang F., Lin L., Bai H., Zhang L., Tang G.Q., Fang H., Mu G.G., Gong W., Liu Z.P. (2011). Raman Spectroscopy for Noninvasive Monitoring of Umbilical Cord Mesenchymal Stem Cells Viability Transitions. Stem Cells in Clinic and Research.

[23] Becker L., Janssen N., Layland S.L., Mürdter T.E., Nies A.T., Schenke-Layland K., Marzi J. (2021). Raman Imaging and Fluorescence Lifetime Imaging Microscopy for Diagnosis of Cancer State and Metabolic Monitoring. Cancers.

[24] Hsu C.-C., Xu J., Brinkhof B., Ye H. (2020). A single-cell Raman-based platform to identify developmental stages of human pluripotent stem cell-derived neurons. Proc. Natl. Acad. Sci. USA.

[25] González-Solís J.L., Martínez-Espinosa J.C., Salgado-Román J.M., Palomares-Anda P. (2014). Monitoring of chemotherapy leukemia treatment using Raman spectroscopy and principal component analysis. Lasers Med. Sci..

[26] Feizpour A., Marstrand T., Bastholm L., Eirefelt S., Evans C.L. (2021). Label-Free Quantification of Pharmacokinetics in Skin with Stimulated Raman Scattering Microscopy and Deep Learning. J. Investig. Dermatol..

[27] Hira M.T., Razzaque M.A., Angione C., Scrivens J., Sawan S., Sarker M. (2021). Integrated multi-omics analysis of ovarian cancer using variational autoencoders. Sci. Rep..

[28] Hu S., Morris I.K., Singh J.P., Smith K.M., Spiro T.G. (1993). Complete assignment of cytochrome c resonance Raman spectra via enzymic reconstitution with isotopically labeled hemes. J. Am. Chem. Soc..

[29] Brazhe N.A., Evlyukhin A.B., Goodilin E.A., Semenova A.A., Novikov S.M., Bozhevolnyi S.I., Chichkov B.N., Sarycheva A.S., Baizhumanov A.A., Nikelshparg E.I. (2015). Probing cytochrome c in living mitochondria with surface-enhanced Raman spectroscopy. Sci. Rep..

[30] Notingher I., Green C., Dyer C., Perkins E., Hopkins N., Lindsay C., Hench L.L. (2004). Discrimination between ricin and sulphur mustard toxicity in vitro using Raman spectroscopy. J. R. Soc. Interface.

[31] Malini R., Venkatakrishna K., Kurien J., Pai K.M., Rao L., Kartha V.B., Krishna C.M. (2006). Discrimination of normal, inflammatory, premalignant, and malignant oral tissue: A Raman spectroscopy study. Biopolymers.

[32] Naumann D. (1998). Infrared and NIR Raman spectroscopy in medical microbiology. Proc. SPIE.

[33] Shetty G., Kendall C., Shepherd N., Stone N., Barr H. (2006). Raman spectroscopy: Elucidation of biochemical changes in carcinogenesis of oesophagus. Br. J. Cancer.

[34] Thomas G.J., Benevides J.M. (1985). An A-helix structure for poly(dA-dT) poly(dA-dT). Biopolymers.

[35] Ruiz-Chica A.J., Medina M.A., Sánchez-Jiménez F., Ramírez F.J. (2004). Characterization by Raman spectroscopy of conformational changes on guanine–cytosine and adenine–thymine oligonucleotides induced by aminooxy analogues of spermidine. J. Raman Spectrosc..

[36] Bik E., Mateuszuk L., Stojak M., Chlopicki S., Baranska M., Majzner K. (2021). Menadione-induced endothelial inflammation detected by Raman spectroscopy. Biochim. Et Biophys. Acta (BBA) Mol. Cell Res..

[37] Benevides J.M., Wang A.H.J., Van Der Marel G.A., Van Boom J.H., Thomas G.J. (1988). Crystal and solution structures of the B-DNA dodecamer d(CGCAAATTTGCG) probed by Raman spectroscopy: Heterogeneity in the crystal structure does not persist in the solution structure. Biochemistry.

[38] Russo V., Candeloro P., Malara N., Perozziello G., Iannone M., Scicchitano M., Mollace R., Musolino V., Gliozzi M., Carresi C. (2019). Key Role of Cytochrome C for Apoptosis Detection Using Raman Microimaging in an Animal Model of Brain Ischemia with Insulin Treatment. Appl. Spectrosc..

[39] Lakshmi R.J., Kartha V.B., Murali Krishna C., Solomon J.G.R., Ullas G., Uma Devi P. (2002). Tissue Raman Spectroscopy for the Study of Radiation Damage: Brain Irradiation of Mice. Radiat. Res..

[40] Chrobak E., Jastrzębska M., Bębenek E., Kadela-Tomanek M., Marciniec K., Latocha M., Wrzalik R., Kusz J., Boryczka S. (2021). Molecular Structure, In Vitro Anticancer Study and Molecular Docking of New Phosphate Derivatives of Betulin. Molecules.

[41] Stone N., Kendall C., Smith J., Crow P., Barr H. (2004). Raman spectroscopy for identification of epithelial cancers. Faraday Discuss..

[42] Ó Faoláin E., Hunter M.B., Byrne J.M., Kelehan P., McNamara M., Byrne H.J., Lyng F.M. (2005). A study examining the effects of tissue processing on human tissue sections using vibrational spectroscopy. Vib. Spectrosc..

[43] Krafft C., Sobottka S.B., Schackert G., Salzer R. (2006). Raman and infrared spectroscopic mapping of human primary intracranial tumors: A comparative study. J. Raman Spectrosc..

[44] Aminuddin A., Ng P.Y., Leong C.-O., Chua E.W. (2020). Mitochondrial DNA alterations may influence the cisplatin responsiveness of oral squamous cell carcinoma. Sci. Rep..

[45] Von Stechow L., Ruiz-Aracama A., Van De Water B., Peijnenburg A., Danen E., Lommen A. (2013). Identification of Cisplatin-Regulated Metabolic Pathways in Pluripotent Stem Cells. PLoS ONE.

[46] Liu H., Michmerhuizen M.J., Lao Y., Wan K., Salem A.H., Sawicki J., Serby M., Vaidyanathan S., Wong S.L., Agarwal S. (2017). Metabolism and Disposition of a Novel B-Cell Lymphoma-2 Inhibitor Venetoclax in Humans and Characterization of Its Unusual Metabolites. Drug Metab. Dispos..

[47] Chacko J.V., Eliceiri K.W. (2019). Autofluorescence lifetime imaging of cellular metabolism: Sensitivity toward cell density, pH, intracellular, and intercellular heterogeneity. Cytom. Part A.

[48] Jamieson L.E., Harrison D.J., Campbell C.J. (2019). Raman spectroscopy investigation of biochemical changes in tumor spheroids with aging and after treatment with staurosporine. J. Biophotonics.

[49] Eastman A. (1987). The formation, isolation and characterization of DNA adducts produced by anticancer platinum complexes. Pharmacol. Ther..

[50] Enoiu M., Jiricny J., Schärer O.D. (2012). Repair of cisplatin-induced DNA interstrand crosslinks by a replication-independent pathway involving transcription-coupled repair and translesion synthesis. Nucleic Acids Res..

[51] Choi Y.-M., Kim H.-K., Shim W., Anwar M.A., Kwon J.-W., Kwon H.-K., Kim H.J., Jeong H., Kim H.M., Hwang D. (2015). Mechanism of Cisplatin-Induced Cytotoxicity Is Correlated to Impaired Metabolism Due to Mitochondrial ROS Generation. PLoS ONE.

[52] Marzano C., Gandin V., Folda A., Scutari G., Bindoli A., Rigobello M.P. (2007). Inhibition of thioredoxin reductase by auranofin induces apoptosis in cisplatin-resistant human ovarian cancer cells. Free. Radic. Biol. Med..

[53] Rybak L. (1999). Dose dependent protection by lipoic acid against cisplatin-induced ototoxicity in rats: Antioxidant defense system. Toxicol. Sci..

[54] Marullo R., Werner E., Degtyareva N., Moore B., Altavilla G., Ramalingam S.S., Doetsch P.W. (2013). Cisplatin Induces a Mitochondrial-ROS Response That Contributes to Cytotoxicity Depending on Mitochondrial Redox Status and Bioenergetic Functions. PLoS ONE.

[55] Sharma A., Smith H.J., Yao P., Mair W.B. (2019). Causal roles of mitochondrial dynamics in longevity and healthy aging. EMBO Rep..

[56] Suhling K., Hirvonen L.M., Levitt J.A., Chung P.-H., Tregidgo C., Le Marois A., Rusakov D.A., Zheng K., Ameer-Beg S., Poland S. (2015). Fluorescence lifetime imaging (FLIM): Basic concepts and some recent developments. Med. Photonics.

[57] Shimolina L.E., Gulin A.A., Paez-Perez M., López-Duarte I., Druzhkova I.N., Lukina M.M., Gubina M.V., Brooks N.J., Zagaynova E.V., Kuimova M.K. (2020). Mapping cisplatin-induced viscosity alterations in cancer cells using molecular rotor and fluorescence lifetime imaging microscopy. J. Biomed. Opt..

[58] Bose P., Gandhi V., Konopleva M. (2017). Pathways and mechanisms of venetoclax resistance. Leuk. Lymphoma.

[59] Rogalińska M. (2002). Alterations in cell nuclei during apoptosis. Cell. Mol. Biol. Lett..

[60] Bower A.J., Sorrells J.E., Li J., Marjanovic M., Barkalifa R., Boppart S.A. (2019). Tracking metabolic dynamics of apoptosis with high-speed two-photon fluorescence lifetime imaging microscopy. Biomed. Opt. Express.

[61] Zbinden A., Carvajal Berrio D.A., Urbanczyk M., Layland S.L., Bosch M., Fliri S., Lu C.E., Jeyagaran A., Loskill P., Duffy G.P. (2020). Fluorescence lifetime metabolic mapping of hypoxia-induced damage in pancreatic pseudo-islets. J. Biophotonics.

[62] Bower A.J., Marjanovic M., Zhao Y., Li J., Chaney E.J., Boppart S.A. (2017). Label-freein vivocellular-level detection and imaging of apoptosis. J. Biophotonics.

[63] Chen T., Guestrin C. (2016). XGBoost: A Scalable Tree Boosting System. arXiv.

[64] Geng R., Harland N., Montes-Mojarro I.A., Fend F., Aicher W.K., Stenzl A., Amend B. (2022). CD24: A Marker for an Extended Expansion Potential of Urothelial Cancer Cell Organoids In Vitro?. Int. J. Mol. Sci..

[65] Fischer F., Birk A., Frenner K., Herkommer A. (2022). FeaSel-Net: A Recursive Feature Selection Callback in Neural Networks. TechRxiv.

[66] Srivastava N., Hinton G., Krizhevsky A., Sutskever I., Salakhutdinov R. (2014). Dropout: A Simple Way to Prevent Neural Networks from Overfitting. J. Mach. Learn. Res..

[67] Kingma D.P., Ba J. (2014). Adam: A Method for stochastic optimization. arXiv.

