# Supplementary material for: Data-Driven Identification of Biomarkers for In Situ Monitoring of Drug Treatment in Bladder Cancer Organoids
Source: Int J Mol Sci. 2022 Jun 23;23(13):6956. doi: 10.3390/ijms23136956 (PMC9266781; doi:10.3390/ijms23136956)
Supplement: Supplementary file 1 [file ijms-23-06956-s001.zip › ijms-1757945-supplementary.pdf]

Supplementary Materials

Table S1. Most relevant features identified in PCA loadings.

Dataset	ν_1 [cm^{-1}]	ν_2 [cm^{-1}]	ν_3 [cm^{-1}]	ν_4 [cm^{-1}]	ν_5 [cm^{-1}]
Nuclei					
RT112	744	1315	1450	1585	1660
BCO	751	1308	1447	1591	1660
UCO	680	1406	1499	1591	1666
Mitochondria					
RT112	1092	1251	1321	1635	1700
BCO	1255	1325	1460	1600	1730
UCO	1138	1242	1317	1451	1658

Table S2. Relative improvement [%] of the FeaSel-Net biomarker retrieval compared to the PCA loadings method (accuracy ACC, sensitivity SEN, and specificity SPE).

Dataset	ACC	SEN	SPE
Nuclei (average)	10.1	18.0	7.1
RT112	13.6	23.9	9.5
BCO	7.2	12.6	5.1
UCO	9.6	17.6	6.6
Mitochondria (average)	5.3	9.1	3.6
RT112	8.3	14.6	5.7
BCO	1.9	3.2	1.4
UCO	5.5	10.1	3.8
All Data	7.3	12.8	5.1

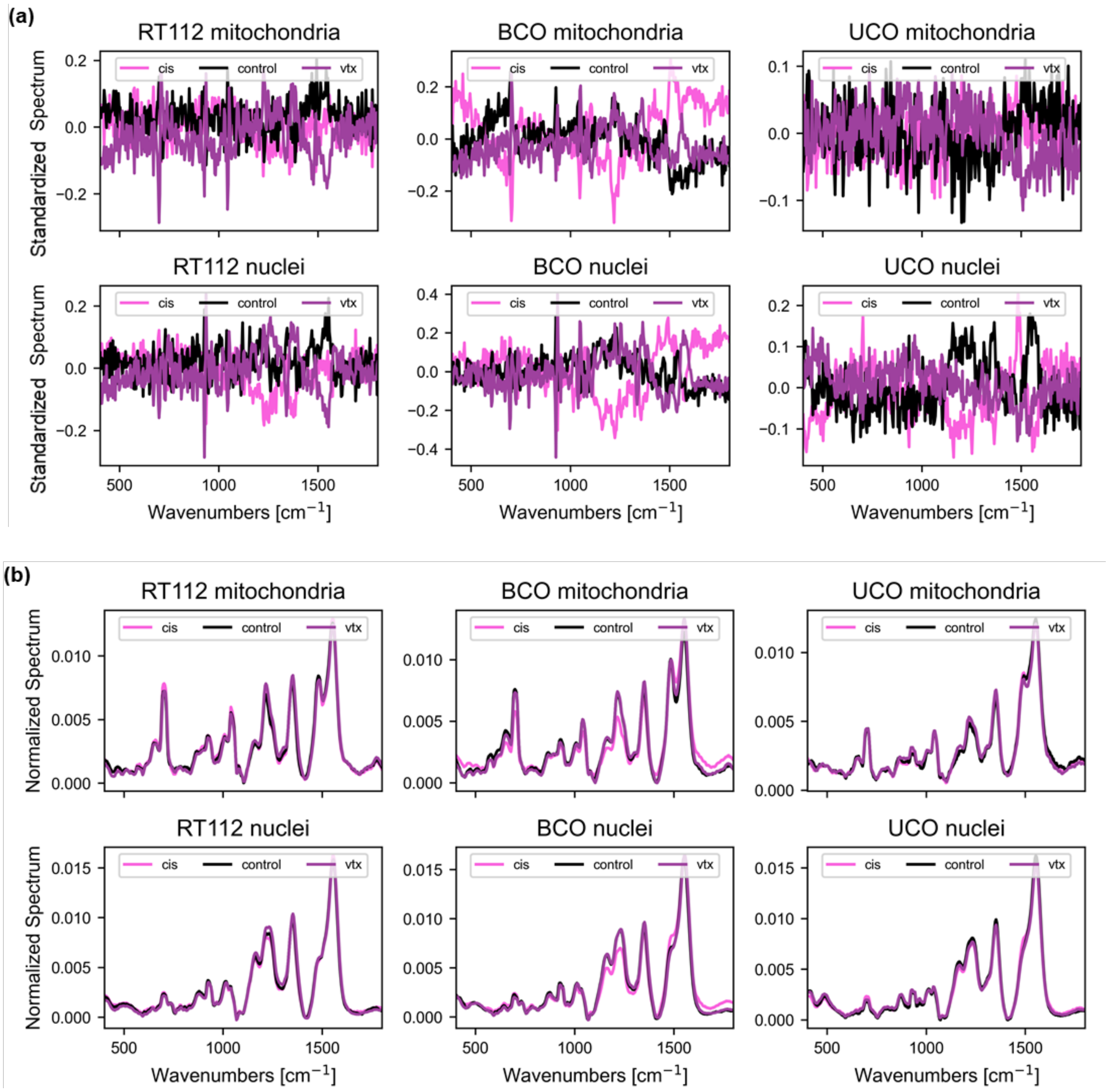

Figure S1. Raman spectra averaged over each class. (a) shows the mean spectra for the standardized spectra along the feature axis whereas **(b)** shows the normalized spectrum along the sample axis.

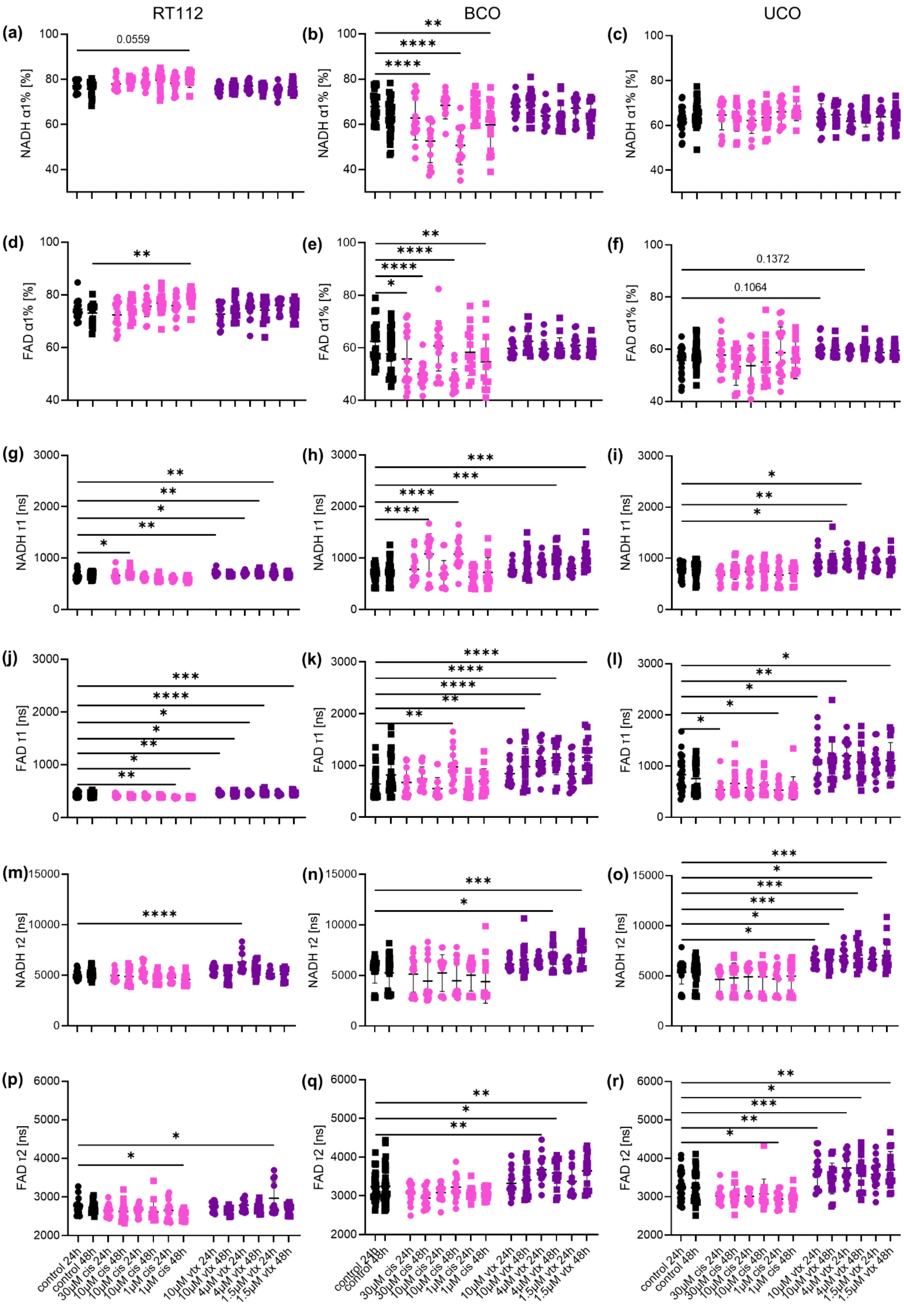

Figure S2. FLIM results for organoids derived from the cell line RT112, BCO and UCO. (a-c) α1% from NADH. **(d-f)** α1% from FAD. **(g-i)** τ1 from NADH. **j-l)** τ1 from FAD. **m-o)** τ2 from NADH. **(p-r)** τ2 from FAD. Data represent average FLIM parameters per organoid, n=15, One-way ANOVA, $p<0.05$.

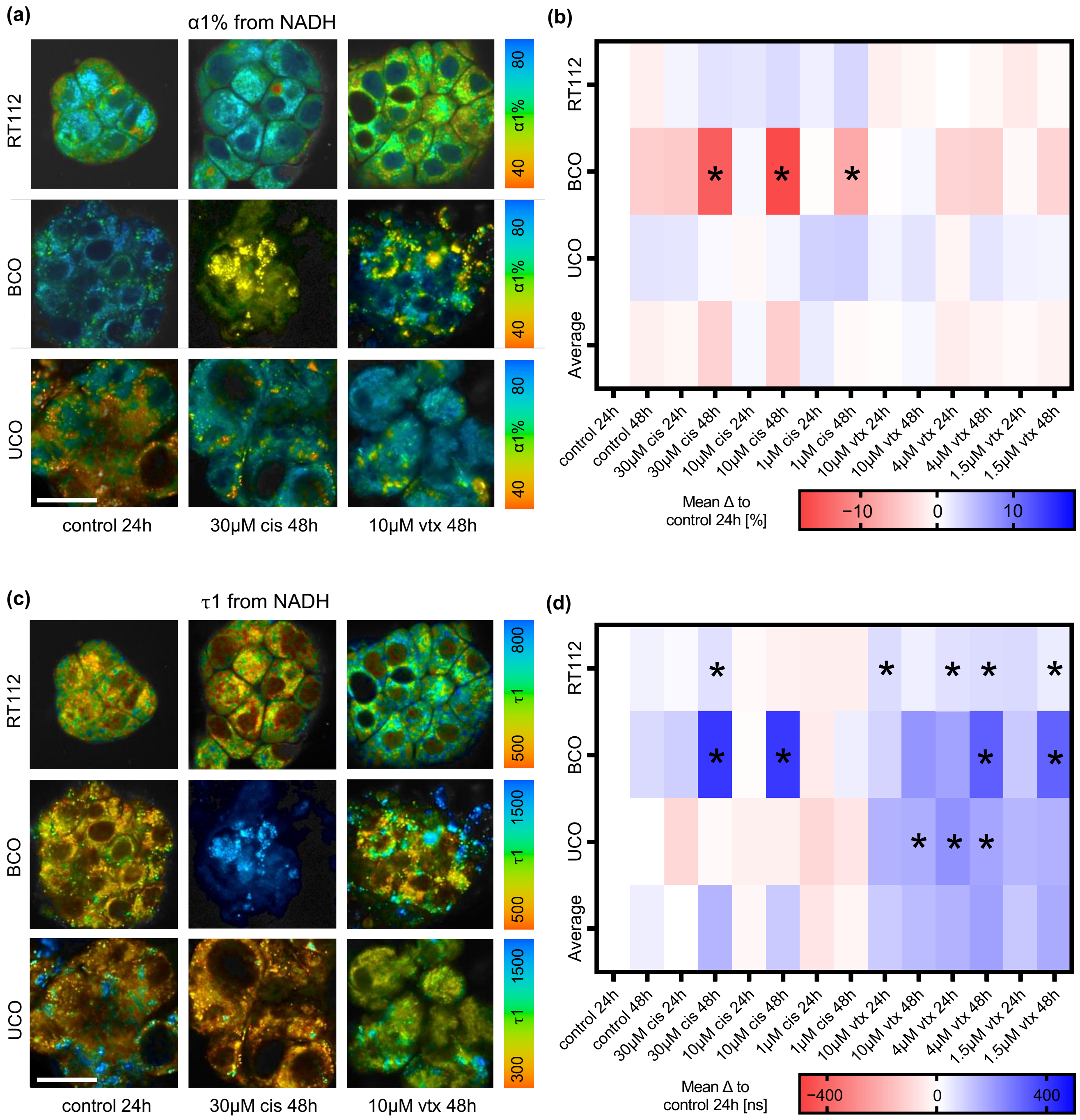

Figure S3. NADH FLIM of bladder cancer organoids. (a) α1% and **(c)** τ1 of RT112, BCO and UCO. Displayed are untreated organoids after 24 hours, 30 µM *cis* treated organoids after 48 hours and 10 µM *vtx* treated organoids after 48 hours. Scale bar: 25 µm. **(b)** Heatmap of mean differences of NADH α1% to the control after 24 hours. **(d)** Heatmap of mean differences of NADH τ2 to the control after 24 hours. One-way ANOVA, n=15, p<0.05.

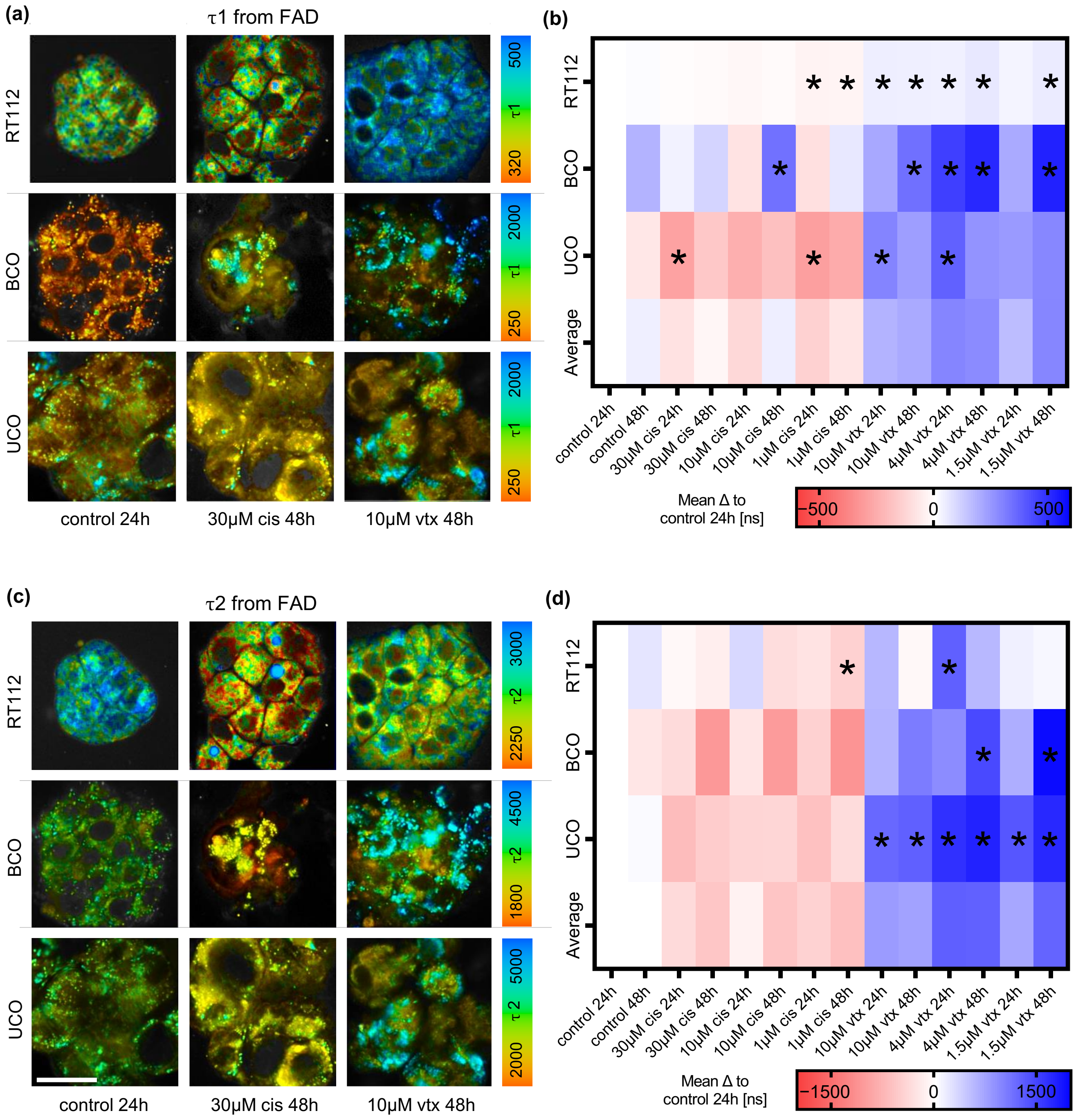

Figure S4. FAD FLIM of bladder cancer organoids. (a) τ1 from FAD and τ2 from FAD **(c)** of RT112, BCO and UCO. Displayed are untreated organoids after 24 hours, 30µM *cis* treated organoids after 48 hours and 10µM *vtx* treated organoids after 48 hours. Scale bar: 25µm. **(b)** Heatmap of mean differences of τ1 from FAD to the control after 24 hours. **(d)** Heatmap of mean differences of τ2 from FAD to the control after 24 hours. Statistical analysis: One-way ANOVA, n=15, $p<0.05$.

Table S3. Information on tumor samples.

Patient	UCO	BCO
Age (years)	85	64
Gender	male	male
Localization	bladder	bladder
Tumor Stage (pT)	pT1	pT2
Tumor Grade (G)	G2	G3
Specimen retrieval	Rinsing urine	Radical cystectomy

Table S4. Parameters for the FeaSel-Net Callback.

Parameter	Symbol	Value
Number of features	n_f	3
Evaluation type	-	accuracy
Threshold value	τ_{acc}	0.98
Threshold decay	σ_{acc}	0.0
Minimum number of epochs	d_{min}	20
Maximum number of epochs	d_{max}	150
Pruning rate	π	0.2
Pruning type	-	exponential
Number of samples per class (eval.)	n_s	10
Normalization of eval. loss	-	min-max norm
Decision metric	-	average
